# Soil Penetration by Earthworms and Plant Roots—Mechanical Energetics of Bioturbation of Compacted Soils

**DOI:** 10.1371/journal.pone.0128914

**Published:** 2015-06-18

**Authors:** Siul Ruiz, Dani Or, Stanislaus J. Schymanski

**Affiliations:** Department of Environmental Systems Science, ETHZ, Zurich, Switzerland; Institute for Sustainable Plant Protection, C.N.R., ITALY

## Abstract

We quantify mechanical processes common to soil penetration by earthworms and growing plant roots, including the energetic requirements for soil plastic displacement. The basic mechanical model considers cavity expansion into a plastic wet soil involving wedging by root tips or earthworms via cone-like penetration followed by cavity expansion due to pressurized earthworm hydroskeleton or root radial growth. The mechanical stresses and resulting soil strains determine the mechanical energy required for bioturbation under different soil hydro-mechanical conditions for a realistic range of root/earthworm geometries. Modeling results suggest that higher soil water content and reduced clay content reduce the strain energy required for soil penetration. The critical earthworm or root pressure increases with increased diameter of root or earthworm, however, results are insensitive to the cone apex (shape of the tip). The invested mechanical energy per unit length increase with increasing earthworm and plant root diameters, whereas mechanical energy per unit of displaced soil volume decreases with larger diameters. The study provides a quantitative framework for estimating energy requirements for soil penetration work done by earthworms and plant roots, and delineates intrinsic and external mechanical limits for bioturbation processes. Estimated energy requirements for earthworm biopore networks are linked to consumption of soil organic matter and suggest that earthworm populations are likely to consume a significant fraction of ecosystem net primary production to sustain their subterranean activities.

## Introduction

The ability of earthworms to move and plant roots to grow through soil greatly affects their capacity to capture resources. Increasing soil compaction gradually decreases the speed and ability of biological growth [1] and movement in soil until a critical threshold is reached in some soils and movement is ceased. Prior to reaching this critical point, both earthworms and plant roots can work to break up soil, thereby ameliorating the negative impacts of soil compaction and improving their own biological habitat [2].

Bioturbation results in a network of channels that promote water infiltration and gas exchange in soil [3], and stimulate microbiological activity and preferential root growth along existing burrows. The stability of biopores is attributed to compaction at their walls [4], the excretion of biopolymers lining the burrows, and to hydrophobicity of mucilage excreted by plant roots [5] or mucus by earthworms [6].

The contributions of earthworms and plant roots to soil structural restoration after compaction [2, 7], and details of the mechanical processes involved have rarely been quantified. Capowiez and Belzunces [8] report that earthworms construct large tunnel networks with lengths ranging from 1 to 2 m per individual earthworm [8]. These burrows involve substantial amounts of displaced soil that may exceed 100 kg m^−2^ ground area per year [7]. Comparatively, plant root growth contributes less to soil biopore construction; estimates of displaced soil mass associated with plant root growth are of the order of 1 kg m^−2^ ground area per year in temperate regions [9].

Relatively few models for the mechanism of soil penetration by plant roots and earthworms have been proposed, such as the model of Greacen and Oh [10] for root growth that balances cell wall pressure and soil pressure applied on root tissue using the cone penetration analogy. This formulation became the standard root growth model ([11], [12], [13], [14]). Greacen *et al*. [4] have shown radiograph images of plant roots growing in a manner that compacts soil around the root circumference rather than at the forefront of the root tip. Hettiaratchi *et al*. [15] observed tissue thickening around the root cap that could promote soil weakening at the cap region resulting in fractures and thus lowering mechanical impedance for root growth. Misra *et al*. [12] have shown evidence that plant roots exert radial pressures notably larger than axial pressures with plant root radial pressures in excess of 2 MPa capable of cracking stiff rigid chalk [12, 16]. Analogously, dry compacted soil fracturing under radial pressures was measured by McKenzie and Dexter [17] in a procedure used to determine threshold radial pressures exerted by earthworms during penetration, showing that earthworms can exert radial pressures slightly above 200 kPa.

The early studies by Dexter [18] on modeling root elongation as an analogue for earthworm penetration were only recently expanded by Dorgan *et al*. [19] and Murphy and Dorgan [20] that proposed models for earthworm burrowing in marine sediments. Dorgan *et al*. [19] used crack propagation models to describe peristalsis during burrowing by earthworms in soft marine sediments. This framework has been shown to provide certain quantitative insights, however, the reliance on fracture mechanics for locomotion in wet soils or soft marine sediments appears questionable, given evidence of slow elasto-plastic deformation during root growth and expansion rates in soft soils, or considering the role of soil rheological properties that mediate other mechanical processes [21]. The primary objective of the present study is to model gradual deformation processes linked with soil penetration by earthworms. The specific objectives are to:

1. Develop a mechanical model to quantify stresses and strains associated with the soil penetration by earthworms and their dependence on soil type, hydration status, earthworm characteristics and geometry
2. Convert mechanical stress-strains to energy equivalents for different soil conditions and earthworm geometries (assuming elasto-plastic soil)
3. Relate these mechanical energy estimates to observed earthworm activity and consumption of soil organic carbon to satisfy energy needs related to soil penetration

We first present theoretical considerations necessary to develop a physically based model for the mechanics and energetics of soil penetration by earthworms that is analogous to plant root penetration models. This is followed by derivation of a simplified analytic expression for minimum earthworm pressure and energy expenditure related to creating a cavity of a given length and diameter under prescribed soil mechanical conditions. Next, we use literature values to estimate soil mechanical conditions from soil water and clay contents and generate estimates of energetic costs and physical constraints on soil penetration in a range of soil conditions. Finally, mechanical and energetic predictions are compared with experimental and empirical evidence from the literature and ecological implications are discussed.

The modeling methodology employs a continuum mechanics approach, thus assuming that penetration occurs through a homogeneous soil medium. This assumption is most applicable in compacted soil, where mechanical constraints to soil penetration are also most severe. Under many natural conditions, soils structure is likely heterogeneous and soil mechanical parameters could be anisotropic. Under these conditions, earthworms and plant roots would likely follow paths of least resistance and greatly reduce energy expenditure for soil penetration.

## Materials and Methods

The symbols used below are listed and described in Table 1.

**Table 1:** Table of symbols used in this study.

Symbol	Definition	SI Unit
*α*	Semi-Apex Insertion Angle	*rad*
*ε*_*r*_	Radial Strain	*m* ⋅ *m* ^−1^
*ε*_*θ*_	Hoop Strain	*m* ⋅ *m* ^−1^
*ϕ*	Friction Angle	*rad*
*G*	Shear Modulus	*Pa*
*k*	Shape Parameter	-
*l*	Distance from Cone tip	*m*
*l*_*b*_	Burrow Length	*m*
*ν*	Poisson’s Ratio	*m* ⋅ *m* ^−1^
*P*	Cavity Pressure	*Pa*
*P*_*L*_	Limit Cavity Pressure	*Pa*
*r*	Radial Depth of Observation	*m*
*R*	Elasto-plastic interface	*m*
*r*_*c*_	Cavity Radius	*m*
*r*_*c*_0__	Initial Cavity Radius	*m*
*r*_*f*_	Final Expanded Cavity Radius	*m*
*σ*_*n*_	Normal Stress	*Pa*
*σ*_*r*_	Radial Stress	*Pa*
*σ*_*θ*_	Hoop Stress	*Pa*
*σ*_*z*_	Axial stress	*Pa*
*s*_*u*_	Soil Strength	*Pa*
*θ*_*m*_	Water Content	*kg* ⋅ *kg* ^−1^
*θ*_*min*_	Residual water Content	*kg* ⋅ *kg* ^−1^
*θ*_*max*_	Saturated water Content	*kg* ⋅ *kg* ^−1^
Θ	normalized water content (actual divided by saturated)	-
*u*	Radial Deformation	*m*
*U*	Strain Energy	*J*
*U*_0_	Strain Energy Density	*J* ⋅ *m* ^−3^

### Modeling penetration-expansion in soil

The focus of the present study is on modeling the mechanics of soil penetration and the formation of macropores primarily by earthworms, in analogy to similar mechanisms applicable to elongating plant roots. We begin by discussing soil penetration by roots and worms concurrently as the equations governing the steady state mechanics of penetration-expansion for the simplified geometries are the same for both. The mechanical model formulation was first explained by Misra *et al*. [12], where the authors described the mechanics of axial and radial growth stresses of plant roots. A similar formulation was applied to earthworms by Mckenzie and Dexter [17]. Dexter [18], [14] unified the description of soil penetration and proposed analogy between roots and earthworms.

**Fig 1:**
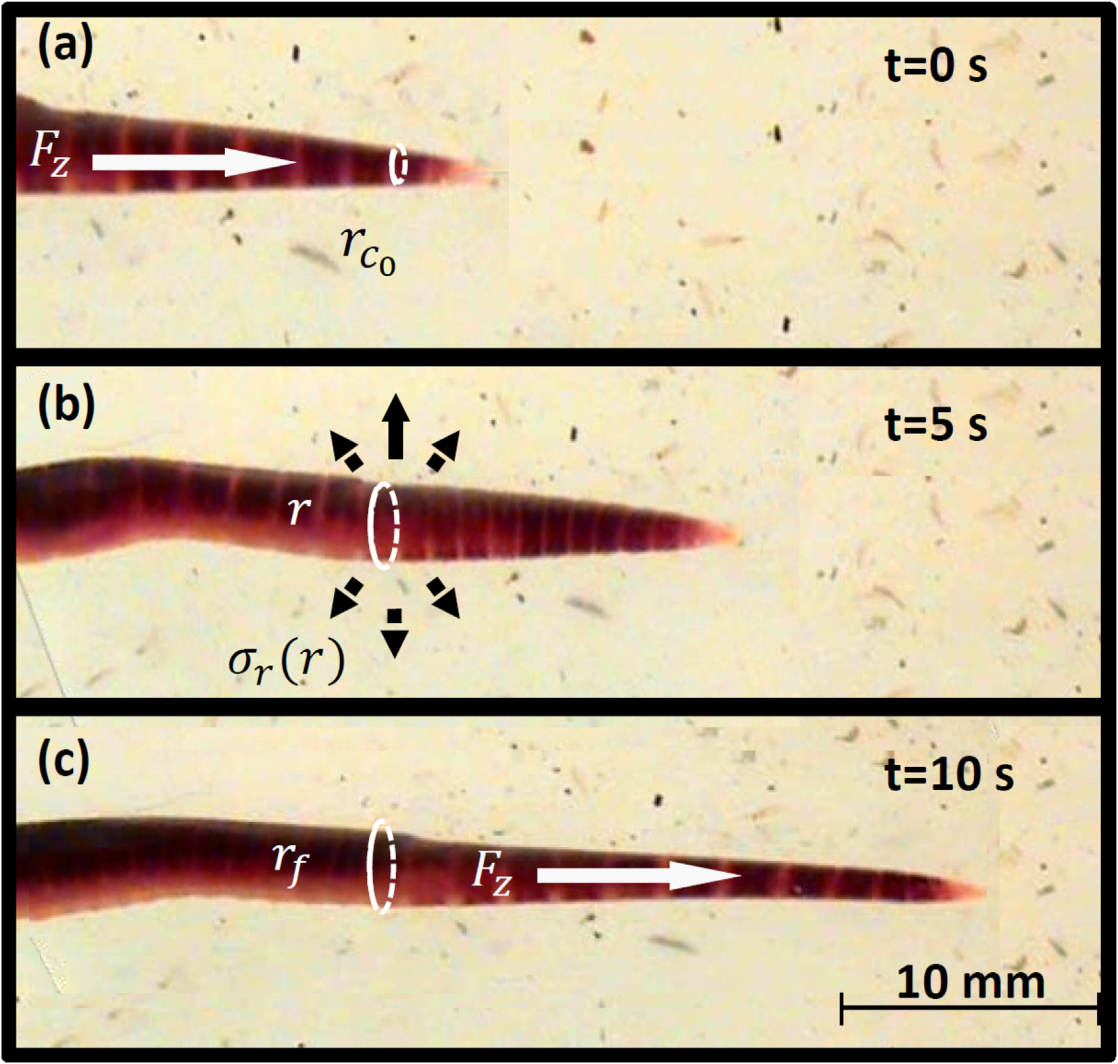
A sequence of images of the front segments of an earthworm moving through agar by a series of penetration-expansion steps. (a) Displays the axial penetration inducing an initial cavity. (b) Illustration of cavity expansion when collecting expanded segments. (c) Further penetration post anchoring processes.

Despite the equivalent formulation of their mechanical soil penetration processes, earthworms and plant roots penetrate soil quite differently. Quillin [22] suggests that earthworm locomotion involves repeated penetration-expansion cycles driven by peristalsis of pressurized colonic fluid. The radial relaxation and contraction of muscles in a wave propagating manner [19, 20] results in localized radial expansion of the earthworm hydrostatic skeleton used for expanding cavities and anchoring during axial penetration. Local radial contraction is also used to elongate earthworm hydrostatic skeleton for more efficient axial penetration of soil [22] as seen in the sequence in Fig 1 (taken in soft agar). Another exclusive feature attributed to earthworms is their ability to ingest soil, however, Quillin [22] reported that earthworm burrows are generally created by displacement of soil rather than ingestion.

Root penetration occurs by tissue growth rather than by peristalsis [10, 23, 24]. The resulting differences originate already at the cellular level, where earthworms utilize muscle fibers for deformation and actuation, thus enabling peristalsis. Plant cells, on the other hand, are surrounded by a stiff cell wall, having yield strengths ranging from 200 to 500 kPa [23, 24]. Root growth occurs when internal cellular pressure within the plant root exceeds the yield strength of cell wall and the soil penetration resistance. This process is facilitated by episodic reorientation of cellulose micro fibrils in cell walls during an enzymatic loosening process, and cell wall thickening during a tightening process following extension [24]. The sequence of cutting, reorientation, and rebuilding can produce pressures in excess of 1600 kPa for extended periods (up to two days) [24].

The most energetically relevant distinction between soil penetration by plant roots and earthworms relates to their penetration rates and penetration pressures. Growth-based soil penetration rates by plant roots range from 0.006 to 0.025 m day^−1^ [13, 25], which is about 20 times slower than earthworm penetration rates. Additionally, plant roots exert pressures of up to 3500 kPa [13, 15, 25–27], 100 times larger than maximal earthworm pressures [13]. Both plant roots and earthworms exhibit larger radial pressures relative to axial pressures during soil penetration, yet both extend predominantly in the axial direction. Misra *et al*. [12] reported axial pressures for plant roots in the range of 200 to 500 kPa relative to radial pressures exceeding 2400 kPa [13]. Keudel and Schrader [28] reported the upper limits of radial pressures exerted by endogeic earthworms in the range between 60 and 195 kPa, whereas axial pressures were limited to the range of 27 to 39 kPa. The capability of plant roots and earthworms to inhabit heavily compacted soils (Capowiez *et al*. [2], Dexter [18]) rely on flexible tissue, reduction of interfacial friction between their bodies and the soil (e.g., mucilage and mucus), and seeking out least mechanically impeding pathways [10, 13, 18].

Bengough and Mullins [13] reviewed mechanical resistances experienced by plant roots during cone penetration and reported 2 to 9-fold higher cone resistances compared to root resistances at similar conditions. These authors have suggested that reduced root-soil friction compared to metal-soil friction [10]could offer an explanation, but mentioned other factors including dynamic effects due to rapid cone insertion rates relative to root growth rates, and radially dominated soil deformation by roots versus axially dominated deformation by a cone penetrometer. Despite differences between penetration by plant roots and cone penetrometers, Atwell [11] stated that penetrometers provide information that correlates well with mechanical impedance experienced by plant roots. Previous models based on pressure balance of a plant root or earthworm rely heavily on cone penetration formulations. Greacen and Oh [10] utilized a formulation describing penetrometer resistance in order to compute the external pressures being applied to the plant root by the soil. More empirical methods of correlating the root growth resistance to the penetration measurements were used by Dexter [14]. Furthermore, Dexter [18] modeled soil penetration by earthworms analogously to plant roots, establishing an indirect link between earthworm penetration and cone penetration.

Motivated by Bishop *et al*. [29], who observed that the pressure required to produce holes in an elastic-plastic medium is proportional to the pressures required to expand a cavity to the same final diameter, we employed a common approach for these linked processes of cone penetration and cavity expansion to describe soil penetration by earthworms. The model utilizes a simple theoretical limit pressure for cavity expansion that translates to axial penetration resistance as mediated by the cone geometry. For simplicity, we consider a steady state continuum elastic-plastic mechanical model. Bishop *et al*. [29] found that for a sufficiently small cone angle, the resisting pressure converges towards the theoretical limiting pressure for cylindrical cavity expansion. Considering that the apex insertion angle of an earthworm and plant root is more acute than that of standardized cone penetrometers, the cylindrical cavity expansion model may be appropriate for describing the process. Bishop *et al*. [29] and Carter *et al*. [30] established the theoretical foundations for large deformation cavity expansion. Assuming a constant proportionality between the plastic and elastic fields around an expanding cavity, the authors showed that cavity pressures at large deformations would converge to a limit pressure depending on the ratio between the soil strength and shear modulus. Consequently, the stress-strain relationship obeys an associated flow law based on a Mohr-Coulomb criterion for all strains within a given radius, and act as a linear-elastic solid material outside of this boundary.

Plastic deformation is a critical process during soil penetration by earthworms in saturated and unsaturated soils. Plastic soil deformation is defined as an irreversible process resulting from cavity pressures exceeding the soil’s yield threshold. Soil plasticity is crucial for the ability of earthworms to exist in soil, otherwise earthworms would constantly need to invest energy to overcome elastic rebound and would not be able to create lasting channels. Earthworms subjected to dry compacted conditions have been reported to struggle in predominantly elastic soils [17].

### Cavity expansion in an elasto-plastic media

**Fig 2:**
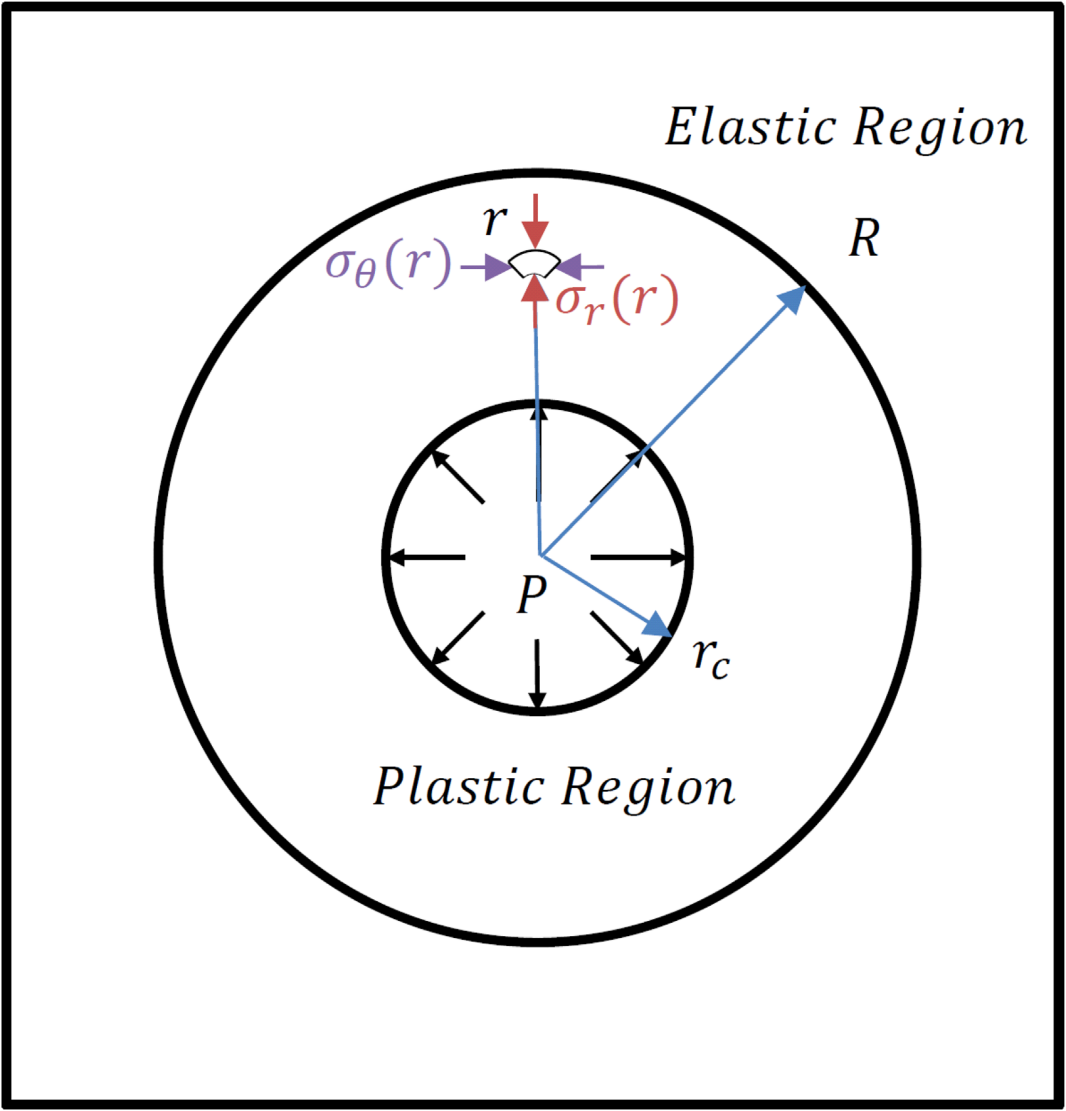
Concept of Elasto-Plastic cavity expansion. Cavity expansion is based on the assumption of a constant ratio between the initial cavity (*r*_*c*_) and a fixed Elasto-Plastic interface (*R*) at a distance proportional to the internally applied cavity pressure (*P*). The stress field propagating into the soil, (*σ*_*r*_, *σ*_*θ*_) depends on the distance from the center (*r*).

At equilibrium, the stress around a cylindrical cavity decays with the radius *r* from the center of the cavity into the surrounding medium (soil) as:

$$\begin{array}{c} \frac{\partial \sigma_{r}}{\partial r}+\frac{\sigma_{r}-\sigma_{\theta }}{r}=0 \end{array}$$ (1)

where *σ*_*r*_ is the radial stress, and *σ*_*θ*_ is the hoop (circumferential) stress (Fig 2). The mechanical transition from elastic deformation to plastic deformation is expressed by the Mohr-Coulomb criterion, relating the difference between the radial and the hoop stresses to the summation of the stresses multiplied by a component of the internal friction and the undrained soil shear strength:

$$\begin{array}{c} \sigma_{r}-\sigma_{\theta }=\left(\sigma_{r}+\sigma_{\theta }\right)sin\left(\varphi \right)+\left(2s_{u}\right)cos\left(\varphi \right) \end{array}$$ (2)

where *ϕ* is the internal angle of friction, and *s*_*u*_ is the undrained soil shear strength. Following Yu [31] and Durban and Fleck [32], we assume frictionless soils, i.e. *ϕ* = 0.

Shames [33] provides strain-displacement relationships in axially symmetric polar coordinates. Given the magnitude of radial soil deformation *u* as a continuous function of the radial distance from the center of the cavity (*r*), the radial strain *ϵ*_*r*_ is defined as the deformation gradient [33 pg 536]:

$$\begin{array}{c} \epsilon_{r}=-\frac{\partial u}{\partial r} \end{array}$$ (3)

The hoop strain *ϵ*_*θ*_ is defined as the ratio between the increase in circumference (2*πr*−2*π*(*r*+*u*)) over the original circumference (2*πr*) [33 pg 536]:

$$\begin{array}{c} \epsilon_{\theta }=-\frac{u}{r} \end{array}$$ (4)

The constitutive law for a continuum material undergoing deformation resulting from an applied stress within the elastic regime is described as [33 pg 526]:

$$\begin{array}{c} \epsilon_{r}=\left(\left(1-\nu \right)\sigma_{r}-\nu \sigma_{\theta }\right)/\left(2G\right) \end{array}$$ (5a)

$$\begin{array}{c} \epsilon_{\theta }=\left(\left(1-\nu \right)\sigma_{\theta }-\nu \sigma_{r}\right)/\left(2Gk\right) \end{array}$$ (5b)

where *G* is the shear modulus of elasticity, *ν* is the Poisson’s ratio of the soil, and *k* is a shape parameter [30], distinguishing between spherical (*k* = 2) and cylindrical (*k* = 1) cavities. Elder [34] solved this problem for incompressible conditions (*ν* = 0.5) considering a spherical cavity i.e. *k* = 2. This is equivalent to assuming that *ϵ*_*r*_ = −2*ϵ*_*θ*_. Adapting Elder’s assumptions and applying them to a cylindrical cavity, we obtain $\varepsilon_{r}=-\varepsilon_{\theta }\Rightarrow \frac{\partial u}{\partial r}=-\frac{u}{r}\Rightarrow u=\frac{C_{1}}{r}$ (where *C*_1_ is an integration constant). Eq 5b simplifies to

$$\begin{array}{c} -2G\frac{u}{r}=-\frac{1}{2}\left(\sigma_{r}-\sigma_{\theta }\right) \end{array}$$ (6)

Substituting $u=\frac{C_{1}}{r}$ in Eq 6 and subsequently Eq 6 into Eq 1 with the boundary condition that $\underset{r\to \infty }{\text{lim}}\sigma_{r}=0$ yields:

$$\begin{array}{c} \sigma_{r}=\frac{2G}{r^{2}}C_{1} \end{array}$$ (7)

Substituting *C*_1_ = *ur* from above and solving for *u* gives:

$$\begin{array}{c} u=\frac{\sigma_{r}}{2G}r \end{array}$$ (8)

In the plastic regime, the radial stress can be estimated by substituting Eq 2 into Eq 1. For a boundary condition of *σ*_*r*_(*r*_*c*_) = *P*, where *r*_*c*_ represents the cavity radius, the radial stress *σ*_*r*_ is defined as:

$$\begin{array}{c} \sigma_{r}=P-2s_{u}ln\left(\frac{r}{r_{c}}\right) \end{array}$$ (9)

At the elasto-plastic interface *r* = *R*, the radial stress yields the value of the undrained soil strength, *σ*_*r*_ = *s*_*u*_, hence the deformation at the elasto-plastic interface is as follows:

$$\begin{array}{c} u\left(r=R\right)=\frac{R}{2G}(P-2s_{u}ln(\frac{R}{r_{c}}))=R(\frac{s_{u}}{2G}) \end{array}$$ (10)

Assuming that the change in the cavity zone ($\pi (r_{c}^{2}-r_{c0}^{2})$) equates to the change in the plastic region(*π*(*R* ^2^−(*R*−*u*)^2^)), we substitute in for Eq 8 ($u(R)=\frac{s_{u}}{2G}R$) and solve for $\frac{R}{r_{c}}$. The relationship explicitly links cavity radius with the plastic radial domain (*r*_*c*_ ≤ *r* < *R*) (Fig 2).

$$\begin{array}{c} {\left(\frac{R}{r_{c}}\right)}^{2}=\frac{G}{s_{u}}(\frac{1-{\left(\frac{r_{c_{0}}}{r_{c}}\right)}^{2}}{1-\frac{s_{u}}{4G}}) \end{array}$$ (11)

Inserting the relationship of Eq 11 back into Eq 10 ($\frac{R}{r_{c}}$), the pressure required to expand a cavity in an elasto-plastic material is expressed as:

$$\begin{array}{c} P=s_{u}(1+ln(\frac{G}{s_{u}}(\frac{1-{\left(\frac{r_{c_{0}}}{r_{c}}\right)}^{2}}{1-\frac{s_{u}}{4G}}))) \end{array}$$ (12)

and under the assumption that the system is incompressible, and $\frac{G}{s_{u}}>>1$, the result yields the limit cavity pressure [30] as *r*_*c*_0__ → 0.

$$\begin{array}{c} P_{L}=s_{u}\left(1+ln\left(\frac{G}{s_{u}}\right)\right) \end{array}$$ (13)

Since plant root and earthworm radii are significantly smaller than standardized cone penetrometer radius, we use data from Bishop *et al*. [29] in conjunction with the limit pressure as a boundary condition. Considering that the deformation is predominantly plastic, we again combine Eq 1 and Eq 2 and apply the boundary condition *σ*_*r*_(*r*_*b*_) = *P*_*L*_ to solve for the radial stress as a function of cavity size:

$$\begin{array}{c} \sigma_{r}\left(r\right)=P_{L}-2s_{u}ln\left(r/r_{b}\right) \end{array}$$ (14)

where *r*_*b*_ (4.45 *mm*) is the radius used by Bishop *et al*. [29] to derive the expression for the cavity limit pressure *P*_*L*_ as described by Carter *et al*. [30]. This formulation facilitates calculation of the required change in cavity pressure for different radii.

### Modeling penetration resistance

**Fig 3:**
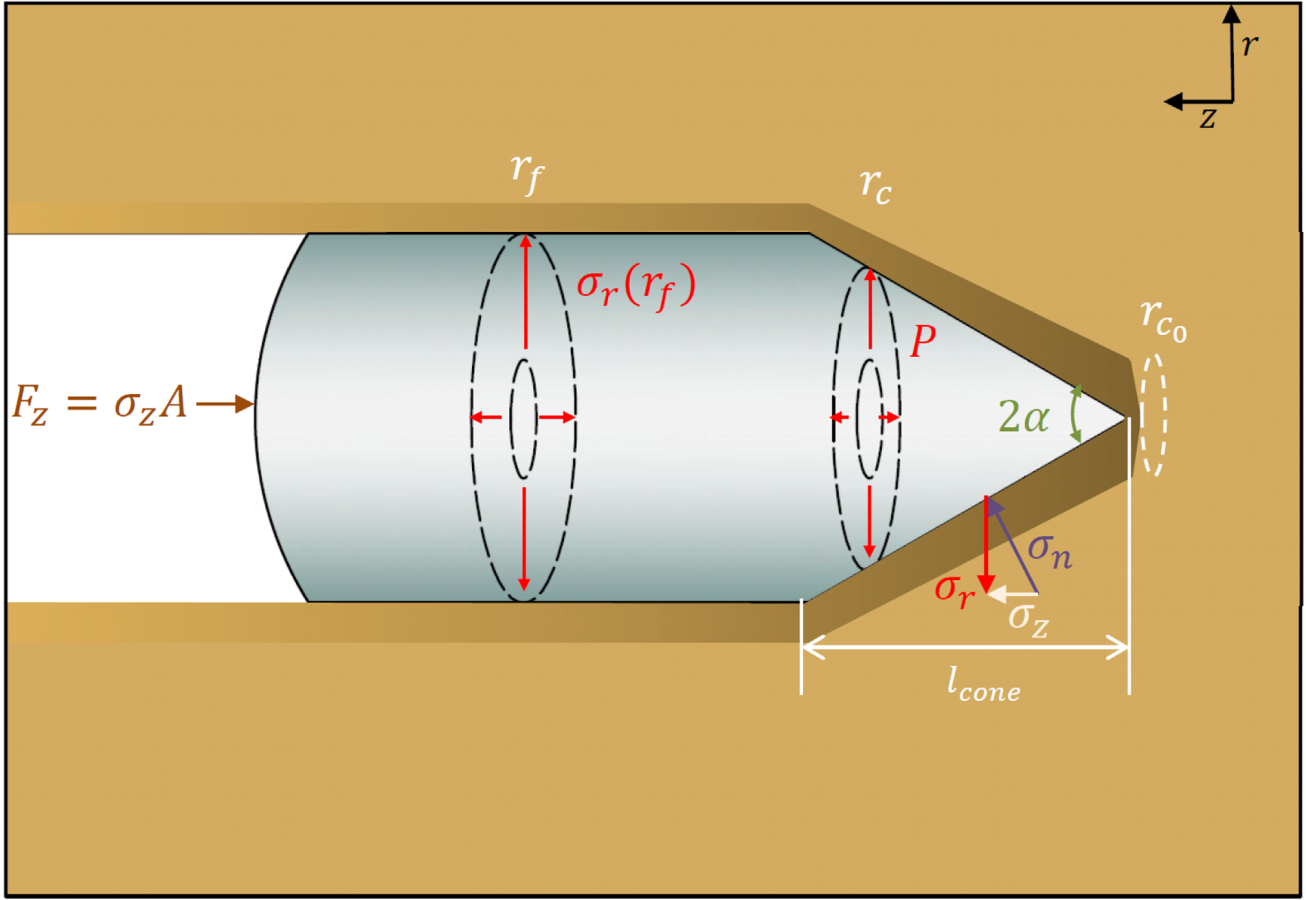
Cylindrical cavity expansion sequentially determines steady state penetration of acute cones. The conical cross section applies a boundary pressure that opens a cavity to some final steady state cylindrical burrow.

Dexter [14, 18], Bengough and Mullins [13] report that plant roots and earthworms penetrate soil in a similar manner as that of sharp penetrometers, deforming the soil cylindrically (see Fig 3). To model soil penetration by earthworms, a cavity expansion based cone penetration model is employed. Yu [31] and Durban and Fleck [32] developed a semi-analytic expression based on cavity expansion for rough and smooth penetration at different angles. This formulation considers the angular effects when neglecting friction. The penetration resistance stress can be expressed as [31, 32]:

$$\begin{array}{c} \sigma_{z}=s_{u}\left(\pi +2\alpha +sin^{-1}\left(m\right)+\frac{D}{2}+m\cot\alpha -\sqrt{1-m^{2}}-1\right)+\sigma_{r} \end{array}$$ (15)

where *α* is the semi-apex cone insertion angle, *m* ∈ [0, 1] is the gauge of roughness where *m* = 0 is lubricated, and *m* = 1 is rough, and

$$\begin{array}{c} D=\frac{\sin\left(\frac{\pi -\alpha }{2}\right)+m\sin\left(\pi -\alpha \right)}{\cos\left(\frac{\pi -\alpha }{2}\right)-\cos\left(\pi -\alpha \right)} \end{array}$$

The penetration resistance force can be determined by integrating the total penetration resistance stress *σ*_*z*_ along the length of the cone for the changing cross sectional area:

$$\begin{array}{c} F_{z}=\{\begin{array}{ccc} 2\pi \int_{0}^{r_{f}}r\sigma_{z}dr, & & 0\leq l<l_{cone}\\ \pi r_{f}^{2}\sigma_{z}, & & l>l_{cone} \end{array} \end{array}$$ (16)

where *r*_*f*_ is the cone base radius, *l*_*cone*_ is the cone length, and *l* is the penetration depth. For *l* ≤ *l*_*cone*_, *l* = *rcot*(*α*) (see Fig 3). For sufficiently long penetration depth where *l* > > *l*_*cone*_, the second expression suffices to account for the effective penetration force.

### Strain energy for cavity expansion in plastic soil—ecological considerations

All energetic costs calculated throughout the text pertain to the mechanical energy required for soil penetration by earthworms (involving plastic deformation and displacement of the soil). The mechanical strain energy is defined as the amount of energy invested to induce deformation based on the following:

$$\begin{array}{c} U=\int_{z_{0}}^{z_{f}}F_{z}dz \end{array}$$ (17)

where *U* is the strain energy of the system, *F*_*z*_ is the penetration resistance force, and *dz* is the change in the penetration depth. By substituting Eq 16 into Eq 17, we obtain:

$$\begin{array}{c} U=\{\begin{array}{ccc} 2\pi cot\left(\alpha \right)\int_{0}^{r_{f}}\int_{0}^{r}\xi \sigma_{z}d\xi dr, & & 0\leq l<l_{cone}\\ \pi r_{f}^{2}\sigma_{z}l, & & l>l_{cone} \end{array} \end{array}$$ (18)

where *σ*_*z*_ is the axial stress, *α* is the semi-apex cone insertion angle, *l* is the axial depth of penetration, *l*_*cone*_ is the axial length of the cone tip, *r* is the cavity radius, *r*_*f*_ is the earthworm base radius, and *ξ* is a dummy variable used for integration, representative of radius. For *l* ≫ *l*_*cone*_, the first term (during insertion) only accounts for a marginal amount of the total strain energy, therefore the second term can be used to approximate the total strain energy related to a penetration depth *l*.

The maximum strain energy density is estimated by dividing the strain energy required to construct small earthworm burrows by their burrow volume, resulting in the following expression:

$$\begin{array}{c} U_{0}=\frac{U(r_{min})}{\pi r_{min}^{2}l} \end{array}$$ (19)

where *r*_*min*_ is taken as the smallest earthworm radius based on ecological parameters, *U*(*r*_*min*_) is the strain energy required for a small cavity, $\pi r_{min}^{2}l$ is the resulting cavity volume. This expression for the strain energy density is used to determine the minimum amount of soil organic carbon content that would meet the mechanic energy demand of earthworm motility.

### Soil mechanical and biophysical properties

**Fig 4:**
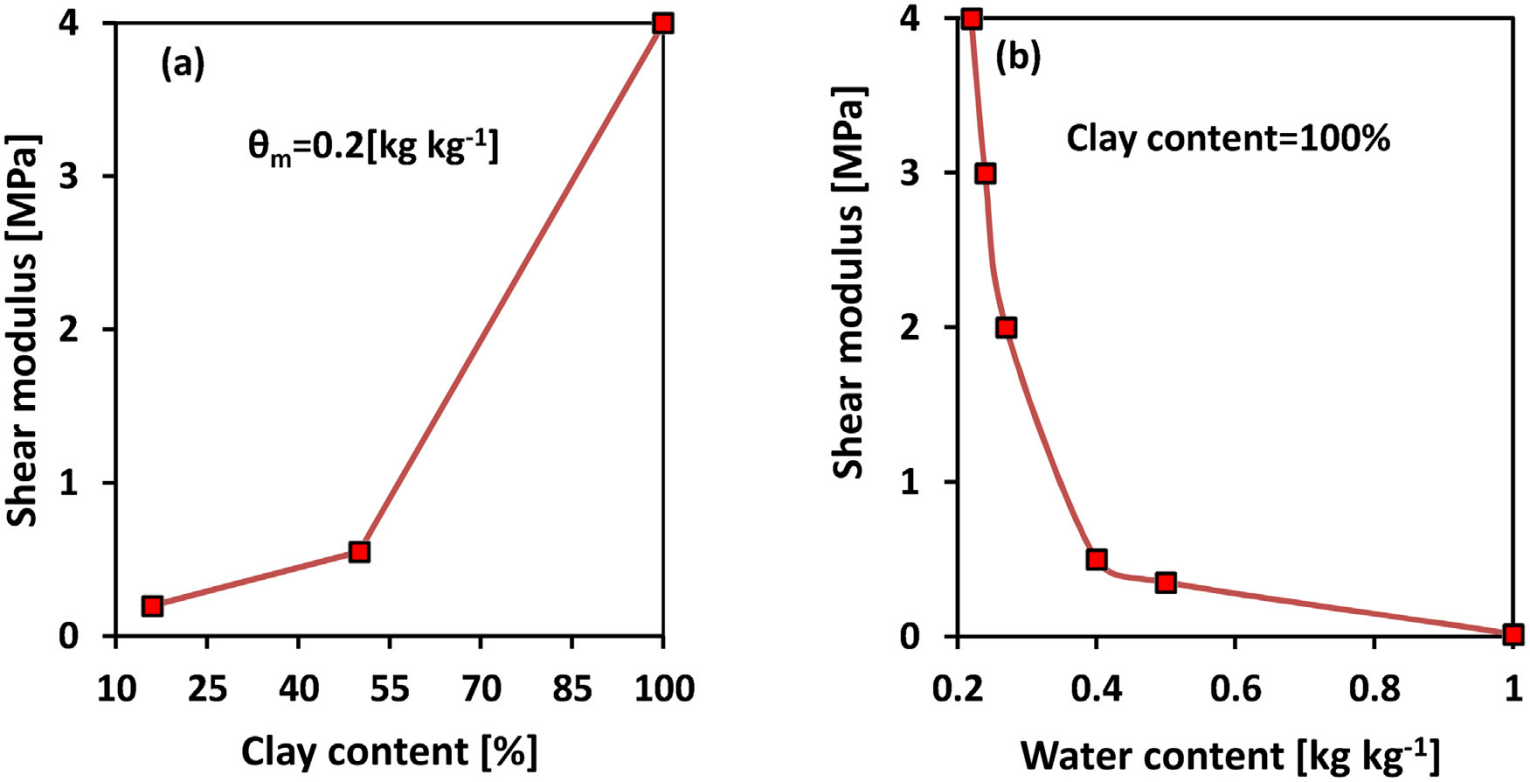
Measured shear modulus values. (a) Different clay contents for fixed water content of 0.2 kg kg^−1^ [21]; (b) different water contents for 100% clay content [21, 38, 39].

The mechanical properties of soils, and mechanical constraints on bioturbation are functions of soil water content, porosity, clay minerals, and other parameters. We explored a range of these soil physical parameters to delineate mechanical stresses and energy costs related to soil penetration by earthworms. A range of data for soil shear strength and elasticity as functions of clay and water contents were obtained from the literature (Table 2). The soil modulus of elasticity was estimated from the clay mechanical properties [38]. To capture the dependence of shear modulus of clay on its water content (Fig 4), these results were expressed in terms of water content and cohesion [39], suggesting that the inferred shear moduli were derived from samples with relatively low water contents. Studies conducted on saturated clays show greatly reduced shear modulus and soil shear strength [21].

**Table 2:** Soil mechanical parameters used in cavity expansion simulations, and their literature sources. *θ*_*m*_: water content, *G*: shear modulus, *s*_*u*_: soil strength.

Clay content %	*θ*_*m*_[*kg* *kg* ^−1^]	*G*[*kPa*]	*s*_*u*_[*kPa*]
15–25	0.20	200 [21]	2–20 [21, 35, 36]
15–25	0.25	70 [21]	0.6 [21]
15–25	0.35	-	5 [35]
15–25	0.40	15 [21]	0.20–20 [21, 35, 36]
40–50	0.20	500 [21]	-
40–50	0.45	150 [21]	4 [21]
40–50	0.70	50 [21]	2 [21]
100	-	5000–17,000 [37]	50–100 [37]
100	0.20	4000 [38]	-
100	0.25	3000 [38]	-
100	0.30	2000 [38]	6 [39]
100	0.40	500 [21]	4 [21]
100	0.50	350 [21]	1.5 [21]

Earthworm physical and ecological parameters were used to determine the mechanical limitations and to estimate the strain energy requirements for soil penetration by earthworms. Observed values of earthworm pressure thresholds were obtained from Newell [40], Keudel and Schrader [28], and McKenzie and Dexter [17], providing insights into the range of soil hydrological conditions that permit earthworms to penetrate soil. The internal earthworm pressure values rarely exceed 230 kPa, with mean values in the range of 60 to 200 kPa [17, 28, 40]. Estimates of earthworm radii were obtained from Vandenbygaart *et al*. [41] and Ehlers [42] with values ranging between 1.0 and 5.5 mm [41], and an earthworm population mean radius of 2.5 mm [42]. Estimates of tunnel lengths were obtained from Capowiez and Belzunces [8]. To estimate annual mechanical energy requirements per unit soil area (or volume) for a typical earthworm community, knowledge of population density and annual penetration rates were needed. Capowiez and Belzunces [8] reported penetration rates for individual earthworms in the range of 0.1 to 0.2 m day^−1^ for measurements over 200 hours. Earthworm population density values were obtained from Daniel [43] and Chan [44] measured at 200 mm soil depths, Fragoso and Lavelle [45], measured at 100 mm soil depths [44] (data are presented in Table 3).

**Table 3:** Earthworm physical parameters. *r*_*f*_: worm radius; *l*_*b*_: tunnel length; $\frac{dl_{b}}{dt}$: penetration rate; *n*: population density; *P*: pressure.

Classification	*r*_*f*_[*mm*]	*l*_*b*_[*mm*]	$\frac{dl_{b}}{dt}[\frac{m}{day}]$	*n*[*ind* *m* ^−3^]	*P*[*kPa*]
Swiss Meadows [43]	-	-	-	700 – 1550	-
Tropical Forest [45]	-	-	-	40 – 4000	-
Agricultural Fields [44]	1 – 5.5 [41, 42]	-	-	300 – 700	-
Lab	-	1000 – 1600 [8]	0.12 – 0.20 [8]	-	3 – 230 [17, 40]

The analysis of penetration expansion was based on models that provided information for determining the amount of mechanical energy required to expand a cavity to radius *r*_*f*_. The range of radii was based on the smallest and the largest earthworm radius in Table 3 (*r*_*f*_ = 1 to 5 mm). Standard mechanical soil properties and their connection to hydration status and clay content were derived from the relations in Ghezzehei and Or [21] (see Table 4). For better comparability between soil types, the hydration status was expressed as normalized water content, defined as

$$\begin{array}{c} \Theta =\frac{\theta -\theta_{min}}{\theta_{max}-\theta_{min}} \end{array}$$ (20)

where *θ*_*max*_ is the highest reported (saturated if available) water content, *θ*_*min*_ is the lowest reported (residual if available) water content, and *θ* is the actual gravimetric water content. The range of reported water content values are tabulated in Table 4. To account for lubrication by biopolymers (e.g. earthworm mucus), the interface between the soil and earthworm was considered to be a frictionless and smooth boundary. The range of apex angles representing earthworm geometries ranged from *α* = 1 to 45^*o*^.

**Table 4:** Input parameters for the Mechanical cavity expansion simulation. *θ*_*min*_: residual water content; *θ*_*max*_: saturated water content; Θ: normalized water content; *G*: Shear modulus; *s*_*u*_: soil strength. (Values marked with an asterisk (*) were extrapolated based on the trend lines presented in [21, 46]).

Clay	*θ*_*min*_	*θ*_*max*_	Θ	*G*	*s*_*u*_
[%]	[*kg*/*kg*]	[*kg*/*kg*]	[−]	[*kPa*]	[*kPa*]
16	0.045	0.4			
			1	15	0.17
			0.44	200	2.30
			0.1*	2386*	40*
50	0.1	0.85			
			1	10	0.6
			0.47	150	4
			0.15*	5668*	38*

### From penetration mechanics to soil displacement energy estimations

Using strain energy per unit length derived from mechanical considerations, estimates of energy demands for bioturbation were derived for different ecological conditions. Using the values of *l*_*b*_ as the length of earthworm channels from Table 3, we calculated mechanical energy costs of soil penetration (per length) and determined the amount of strain energy required for incremental soil penetration. In conjunction with data regarding earthworm penetration rates and earthworm population densities in various ecological systems (Table 3), we estimated the amount of mechanical energy transferred to the soil by a population of earthworms. For simplicity, we consolidated reported vertical distributions of earthworm radius to a single mean value of 2.5 mm, assuming that the value does not vary too much in the top half meter of soil [42]. The estimated energetic costs of soil penetration were translated to equivalent soil organic carbon consumption by earthworms, using a conversion coeeficient of 0.0484 g_*carbon*_ J^−1^ [7].

### Model evaluation—comparison to numerical and empirical data

Predictions by the analytical model of cavity expansion (Eq 12) were first evaluated in comparison with numerical calculations for the same conditions, and then with experimental results. A plane strain steady state finite elements model (FEM) was constructed using COMSOL [47]. The finite element model simulated deformation driven cavity expansion in an incompressible elastic-perfectly plastic medium with boundary walls infinitely far from the expanding cavity. Both the finite element model and the analytic model were compared for 9-fold expansion from an intial radius.

Additional comparisons were performed with an explicit cone penetration model developed by Walker and Yu [48]. Their model uses an adaptive finite element remeshing algorithm in order to simulate the actual motion and geometry of soil penetration directly rather then with cavity expansion. The analytic solution expressed in Eq 15 was used for a cone roughness of *m* = 1 and a semi apex angle of *α* = 30^*o*^. Both models assume *s*_*u*_ = 10 kPa and *G* = 1000 kPa (taken from Walker and Yu [48]).

The model was lastly tested against experimental penetration data of Kurup *et al*. [49], where two replicates of a silty clay (50% Kaolinite and 50% Edgar fine sand) were measured to have soil strengths and shear moduli of a) *s*_*u*_ = 65 kPa and *G* = 567 × *s*_*u*_ and b) *s*_*u*_ = 40 kPa and *G* = 150 × *s*_*u*_ under saturated conditions. We assumed a friction effect of *m* = 1, semi apex angle of 30^*o*^, and utilized the final radial stresses for cone radii of 5.64 and 6.36 mm associated with the cone designs of the miniature piezocone penetrometer and minature quasi-static cone penetrometer respectively [49].

### Comparison to crack propagation model

Results from the present model were also compared with data provided by Dorgan *et al*. [50], who assumed that earthworms penetrate sediments by crack propagation and estimated energy requirements for soil penetration using linear elastic fracture mechanics. Estimations of energy requirements for soil penetration by crack propagation were conducted using linear elastic fracture mechanics (LEFM) utilizing an energy formulation defined by Dorgan *et al*. [50]:

$$\begin{array}{c} U=K_{Ic}^{2}\frac{\left(1-\nu \right)}{2G}lr_{f} \end{array}$$ (21)

where *K*_*Ic*_ is the fracture toughness, *ν* is the Poisson’s ratio, *G* is the shear modulus, *l* is considered to be the distance over which the crack grows, and *r*_*f*_ is considered to be the width of the crack. For simplicity, this study assumes that the width of the crack is the same size as the radius of the earthworm penetrating the soil, and the length that the crack grows is equal to the depth of a given earthworm tunnel. For comparison with our model, we assumed a 1 m long penetration depth with a radius of 1.2 mm [50].

**Fig 5:**
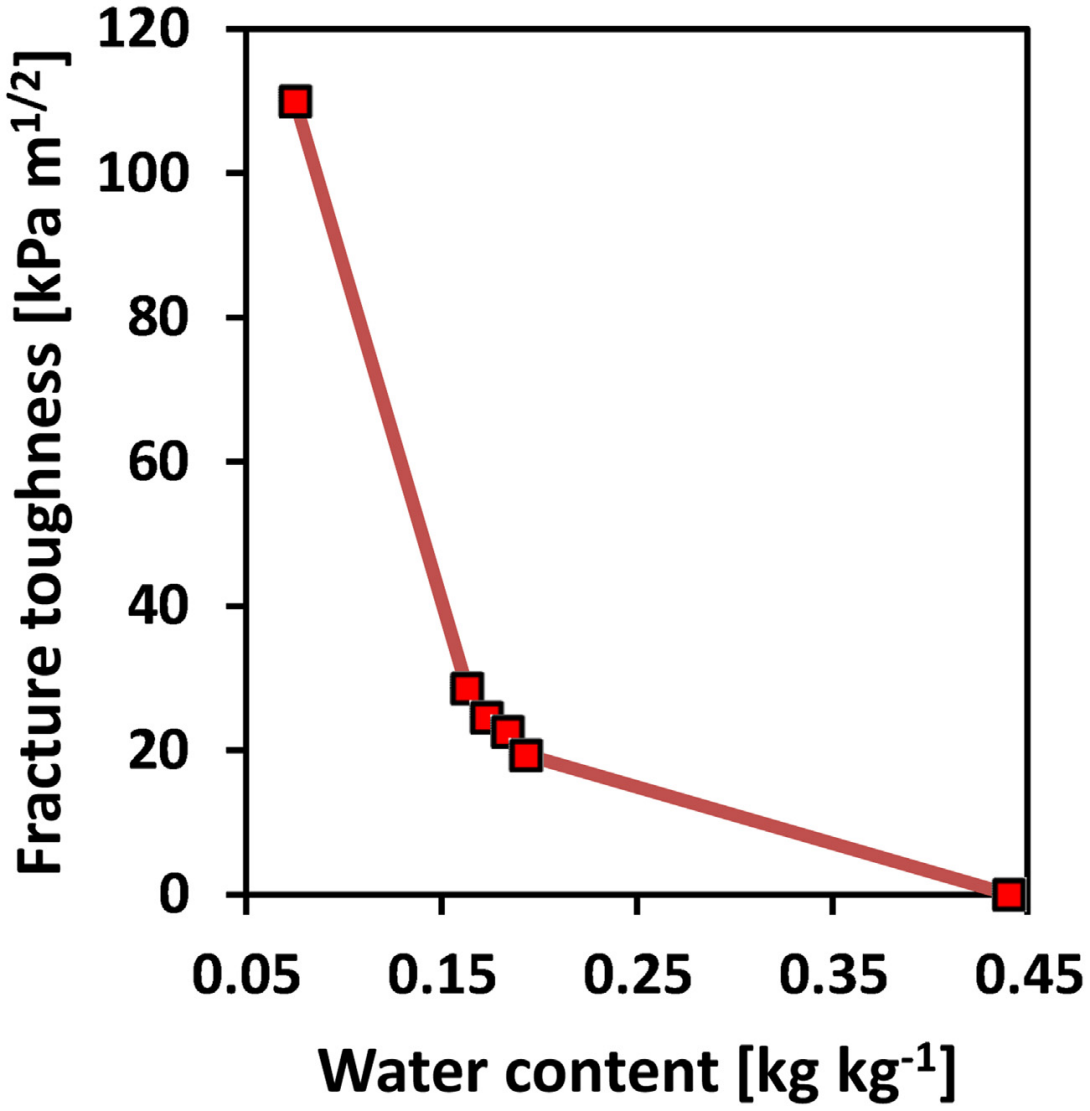
Fracture toughness vs water content. [50–52]. Continuous curve was plotted through the data points in order to approximate fracture toughness values at different water contents.

The fracture toughness changes as a function of soil water content. Values for fracture toughness for lower water contents were collected from Hanson *et al*. [51] and Wang *et al*. [52]. For a soil with clay content of 15–25% and saturated conditions (water content of *θ*_*m*_ = 0.44 kg kg^−1^), the mechanical shear modulus is equivalent to that of gelatine used in the study by Dorgan *et al*. [50] (*G* = 1.4 kPa). The fracture toughness parameters were fit to a continuous curve plotted against water content in order to estimate mechanical energy investments for a wider range of water contents (seen in Fig 5).

## Results

### Model evaluation—comparison to numerical and empirical data

**Fig 6:**
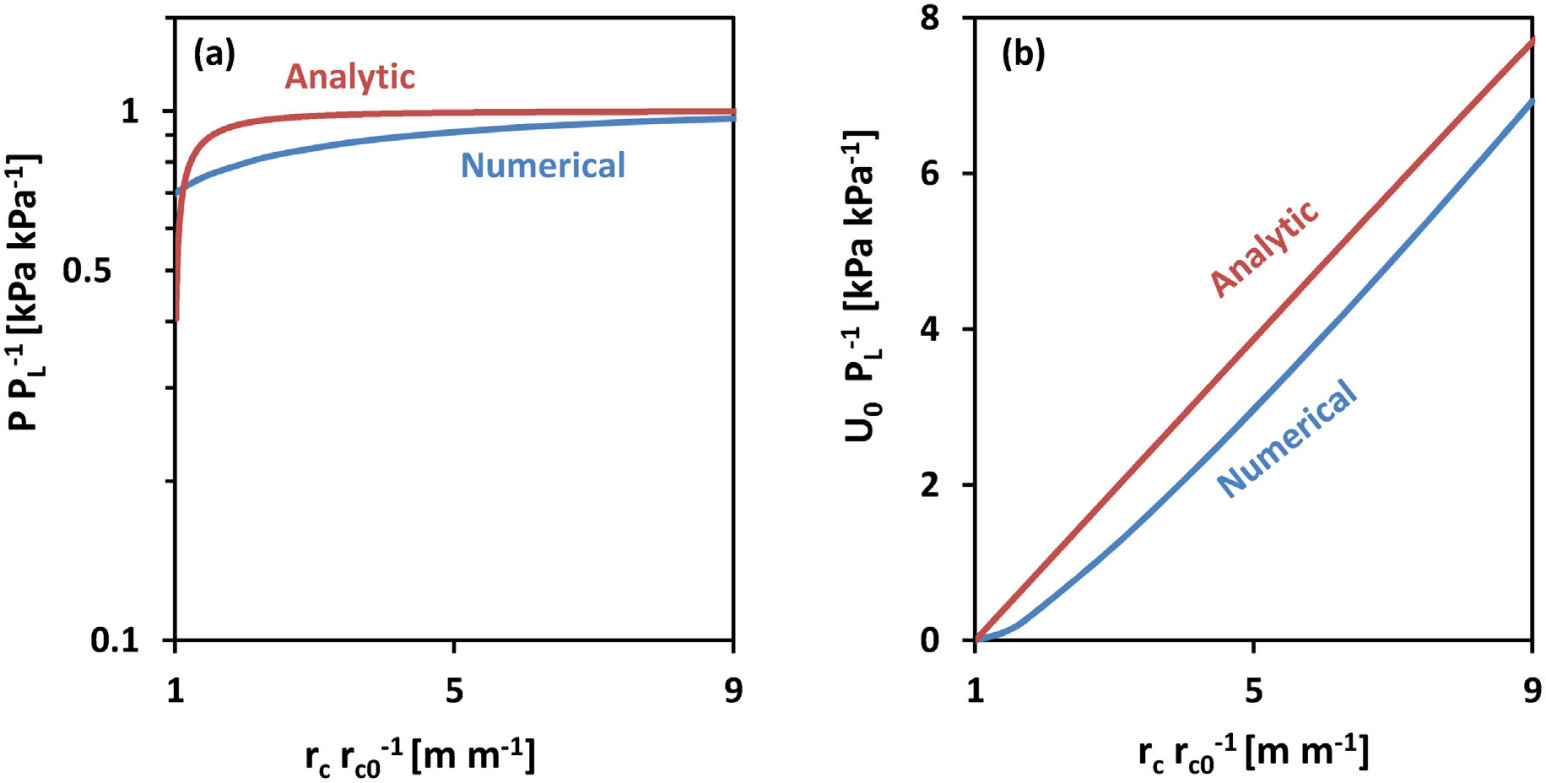
Analytical cavity expansion model *vs*. Finite Element cavity expansion model. (a) Relative cavity pressure *vs*. relative cavity radius; (b) strain energy density scaled by limit pressure *vs*. relative cavity radius scaled by initial radius. For both models, changes in soil mechanical parameters only changed the magnitude of *P*_*L*_. Both analytic and numerical models showed close magnitudes of *P*_*L*_. The discrepancy between the strain energy density values was measured as: $\frac{{\left\|U_{0,NUM}-U_{0,ANA}\right\|}_{\infty }}{{\left\|U_{0,NUM}\right\|}_{\infty }}=0.11$.

The results depicted in Fig 6 illustrate that the limit pressures predicted by the analytic model and the finite element COMSOL model converge for large final cavity radii compared to initial radii, given same soil strength and shear modulus. Note that $PP_{L}^{-1}$ is the ratio of cavity pressure to limit pressure, and the limit pressure is invariant with respect to cavity radius [30].

The results show that the convergence to the limit pressure occurs at a larger radial deformation in the numerical solution than predicted by the analytic solution. However, the difference between strain energy densities for the analytic solution and the numerical solution were less than 10% at 9-fold deformation (Fig 6 (b)).

**Fig 7:**
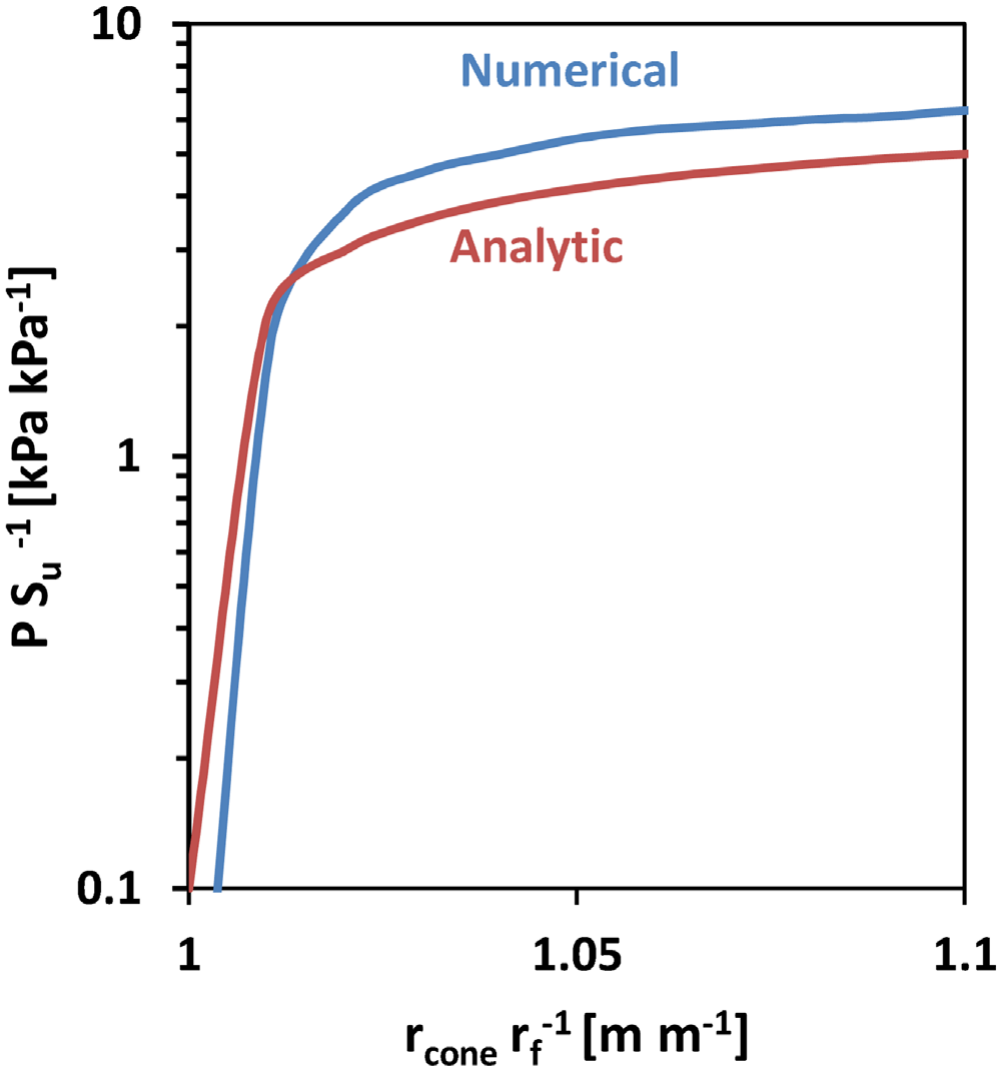
Comparison of analytical cavity expansion based model with an adaptive finite element explicit penetration model (Walker and Yu [48]). Comparison is drawn between the relative penetration stress vs the radial strain. Penetration stress is scaled by the shear soil strength. The discrepancy between the pressure values over two orders of magnitude never exceeds $\frac{{\left\|P_{NUM}-P_{ANA}\right\|}_{\infty }}{{\left\|P_{NUM}\right\|}_{\infty }}<0.2$

The penetration stress versus radial strain was compared between the cavity expansion based cone penetration model and the geometrically explicit adaptive FEM cone penetration model developed by Walker and Yu [48]. The comparison suggests that the simplifying assumptions employed in the analytical model introduced a relative error of less than 20% (Fig 7).

**Fig 8:**
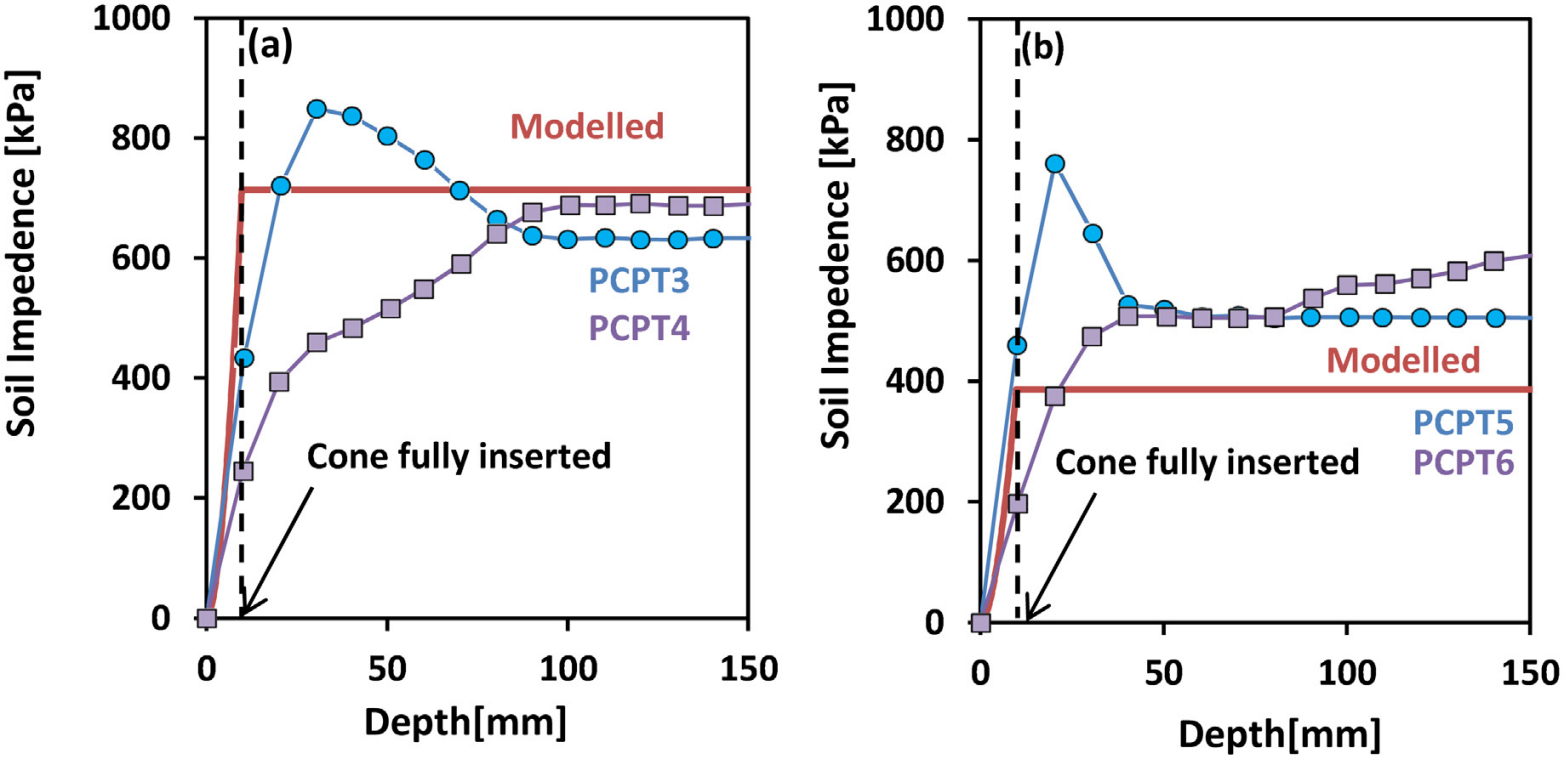
Soil mechanical impedance to cone penetration for different soil mechanical properties and cone types. Two replicates of a silty clay were measured to have (a) *s*_*u*_ = 65*kPa* and *G* = 567 × *s*_*u*_; (b) *s*_*u*_ = 40*kPa* and *G* = 150 × *s*_*u*_. Experimental data correspond to two tests conducted with duplicate cones of the same geometry but subtle physical design differences [49]: miniature piezocone penetrometer (PCPT4 and PCPT6) and miniature quasi-static cone penetrometer (PCPT3 and PCPT5). Data points were obtained from [49] with the dashed lines denoting the positions when the cone was fully inserted.

Finally, the soil impedance predicted with the present penetration-expansion model was compared with experimental impedance results reported by Kurup *et al*. [49] down to a soil depth of 150 mm (seen in Fig 8). The comparison of the simulated stresses and the experimental data reveals an error between measured and predicted soil mechanical impedance of about 8–18% for the first test, and 20 to 35% for the second test. The discrepancy towards the tip could be related to dynamic effects that are not properly accounted for in the current steady state solution.

### Simulated forces and stresses during penetration-expansion in soil

**Fig 9:**
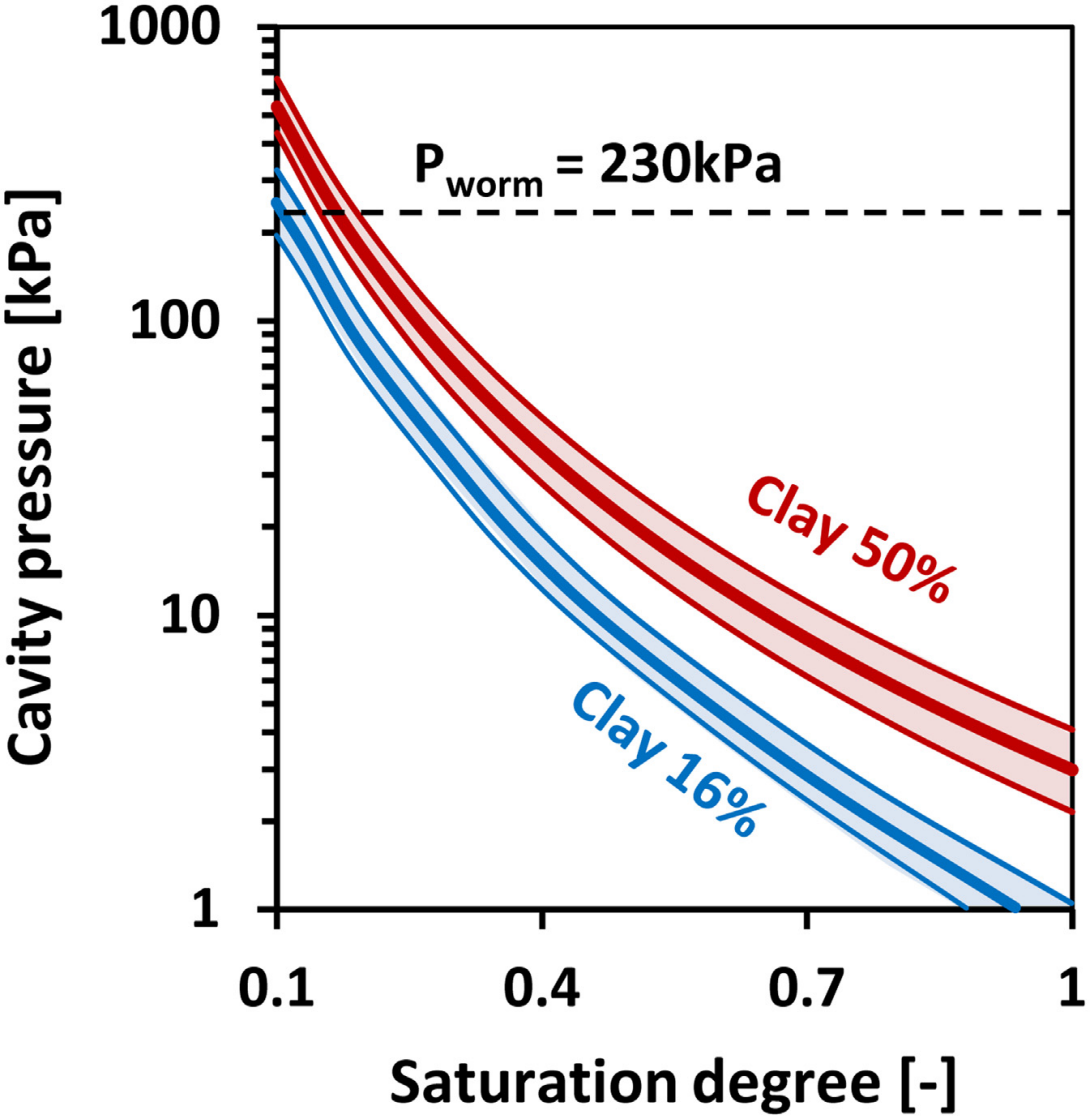
Predicted cylindrical cavity pressure as a function of saturation for two clay contents (16 and 50%) and for a range of cavity radii (1 to 5 mm). The blue and red curves denote soils with clay contents of 16% and 50% respectively, with the hydro-mechanical correlations presented in Table 3. Thick red and blue lines refer to earthworm radius of 2.5 mm, while the enveloping curves represent radii between 1 and 5 mm.

The internal earthworm (or root) pressures required for cavity expansion or for penetration of new soil volumes were computed for various cavity radii, apex insertion angles and combinations of hydration and soil properties listed in Table 2. The results in Fig 9 illustrate that the pressure required for cavity expansion increases for drier soil and reaches the intrinsic maximum pressure (that an earthworm can exert) at normalized water content of roughly 0.1 and 0.2 for soils with clay contents of 16% and 50% respectively.

**Fig 10:**
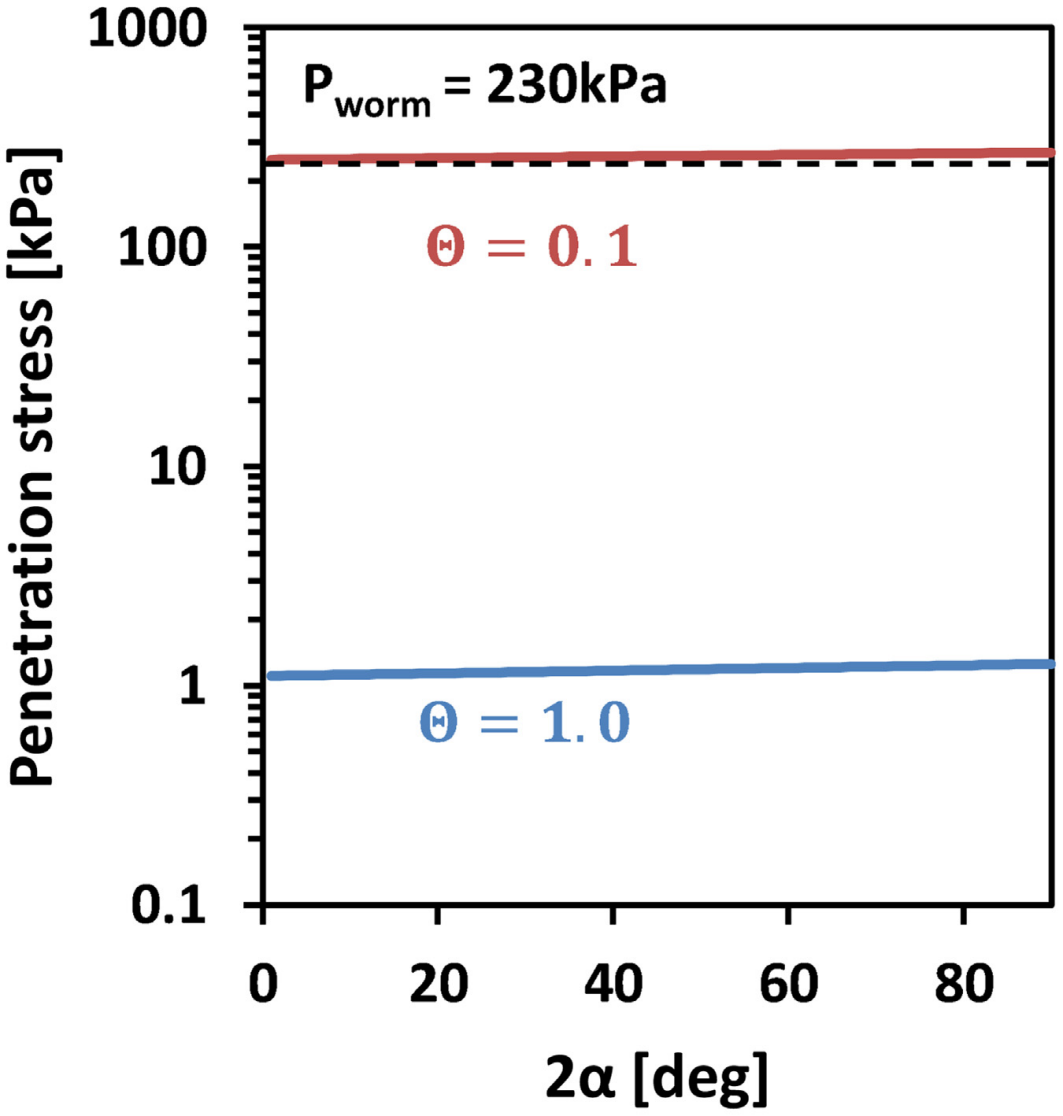
Maximum penetration resistance stress *vs*. cone apex angles for different normalized water contents with a base radius of 2.5 mm at 16% clay content. Simulations were conducted for normalized water contents of 0.1, and 1.0 at a soil clay content of 16%. Soils with larger clay content display similar mechanical behavior at larger normalized water contents.

The exact “cone” geometry has only a small influence on the penetration and cavity expansion pressures. Fig 9b illustrates the maximum penetration resistance pressures for varying semi apex angles for the same moisture contents. The simulations were conducted assuming a frictionless (*m* = 0) interface between the penetrating object (earthworm or plant root) and the soil. Soil drying (reduced water content) increases cavity limiting pressure and with it the penetration resistance. The results in Fig 10 were computed for soils with a clay content of 16%, but similar trends were obtained for soils with 50% clay content (slightly higher mechanical resistance values). In summary, the penetration pressures increase with increasing clay content, decrease with increasing water content, decrease (slightly) with increasing radius, and slightly increase with increasing semi-apex angle.

**Fig 11:**
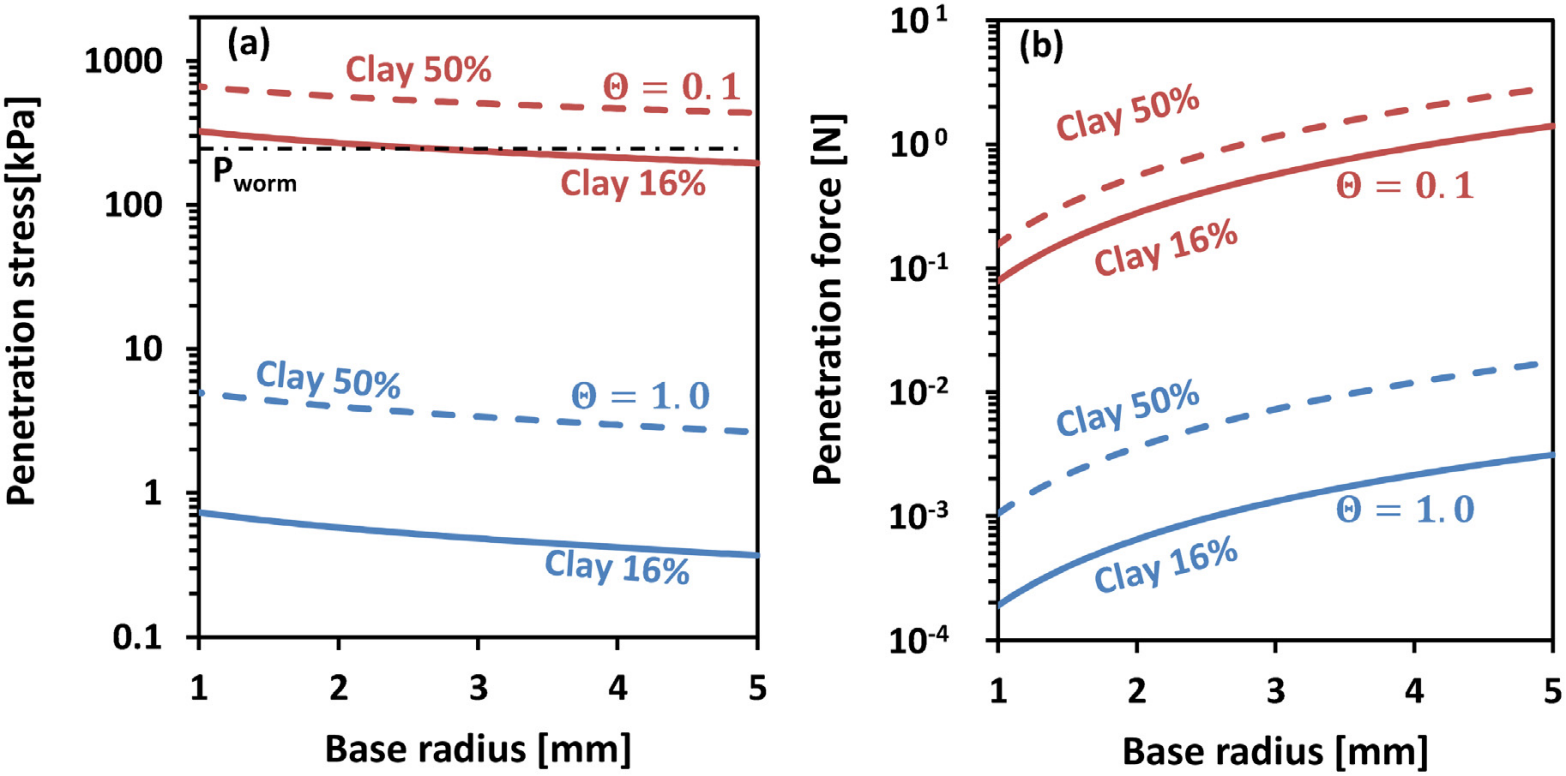
Penetration stress and resistances as a function of base radius. (a) Penetration stress; (b) penetration resistance. Dashed curves denote soils with clay contents of 50%, while solid curves denote clay contents of 16%. Penetration stress (a) decreases for increasing base radius for fixed soil mechanical properties. In contrast, for the same penetration stresses, penetration resistance (measured as axial force) increases with increasing base radius.

Cone penetration resistance in terms of stresses and penetration resistive forces for different earthworm and plant root radii are depicted in Fig 11. Fig 11 (a) shows that the penetration stress decreases (slightly) with increasing radius. In contrast, the penetration force increases with cone radius (Fig 11 (b). The interplay between penetration (resistive) force and stress will be elaborated in the discussion section, in the context of estimating energy costs of bioturbation.

### Strain energy of cavity expansion in plastic soil—ecological considerations

**Fig 12:**
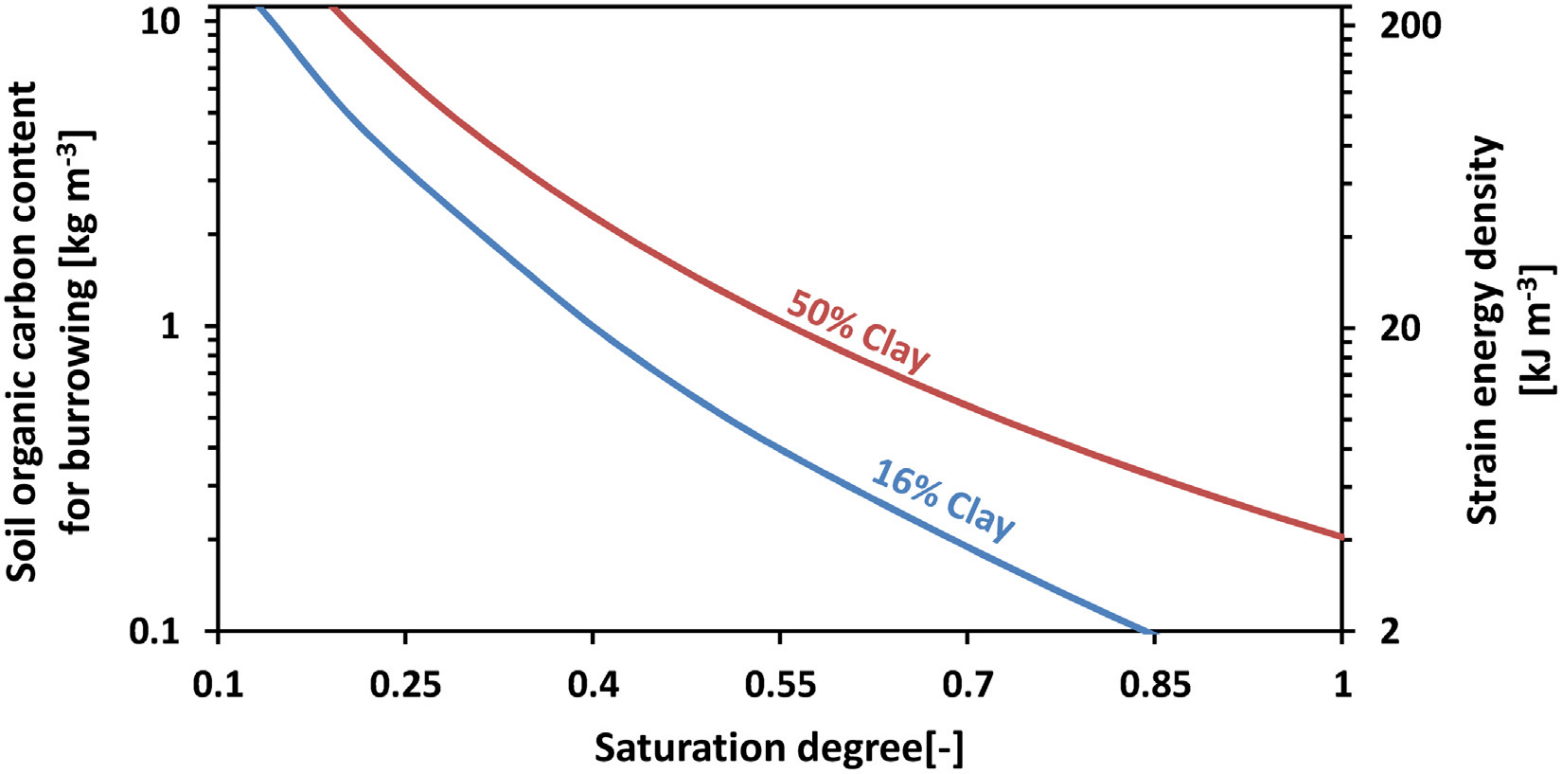
Required soil organic carbon content and maximum strain energy density as a function of normalized water content for clay contents of 16% and 50%. Analysis is based on the steady state mechanical model to determine strain energy. For given soil organic carbon content, one can determine the range of normalized soil moistures under which the energetic demands of displacing a volume of soil are less than the energy stored in the soil organic carbon in the same volume (range to the right of the respective line in the figure). This neglects any rate dependent effects. Note that mechanical energy is mapped to soil organic carbon content using a factor of 1.2/24.8 kg kJ^−1^ [7], and the tick marks on the left vertical axis were spaced in order to align with those on the right vertical axis.

Estimates of bioturbation strain energy were computed for different clay contents and normalized water contents, and for radii in the range of 1 to 5 mm, as listed in Table 2. The strain energy density was estimated from strain energy values associated with the minimum earthworm radius of 1 mm. Fig 12 depicts the change in strain energy density as a function of clay content and water content. Applying a conversion coefficient of 0.0484 g_*carbon*_ J^−1^ to translate energy requirement to soil organic carbon requirement [7], we estimate minimum soil organic carbon (SOC) contents required to support penetration by earthworms in soils with different clay and water contents (Fig 12). The strain energy (and required SOC) decreases with increasing water content, and increases with increasing clay content.

To estimate the annual carbon consumption of earthworm communities, we use published examples of earthworm abundances in different ecosystems (Table 3). For a soil with 16% clay content and normalized water content of 0.4 (representing field capacity [53]), the strain energy density was calculated to be 24.1 kJ m^−3^. For an average earthworm of 2.5 mm radius, the energy required to create a 1 m long tunnel would amount to 24.1 kJ m^−3^ × *π* × (2.5 × 10^−3^ m)^2^ × 1 m = 4.7 × 10^−4^ kJ. Assuming a mean penetration rate of 0.1 m day^−1^(or 36.5 m year^−1^) (Table 3), we estimate an annual mechanical work of 17.2 J year^−1^ per earthworm, which is equivalent to the energy contained in 0.8g of SOC. Assuming a typical earthworm population density in a paddock of 300 individuals per m^3^ soil in the top 0.2 *m* of soil (Table 3) and the same penetration rate of 0.1 m day^−1^ for each individual, we estimate a minimum consumption of 8 × 10^ − 4^ kg year^−1^ × 300 ind m^−3^ × 0.2 m = 0.05 kg year^−1^ soil organic carbon per *m* ^2^ surface area to cover the energy expenditure for soil penetration by the whole earthworm community.

### Comparison to crack propagation model

**Fig 13:**
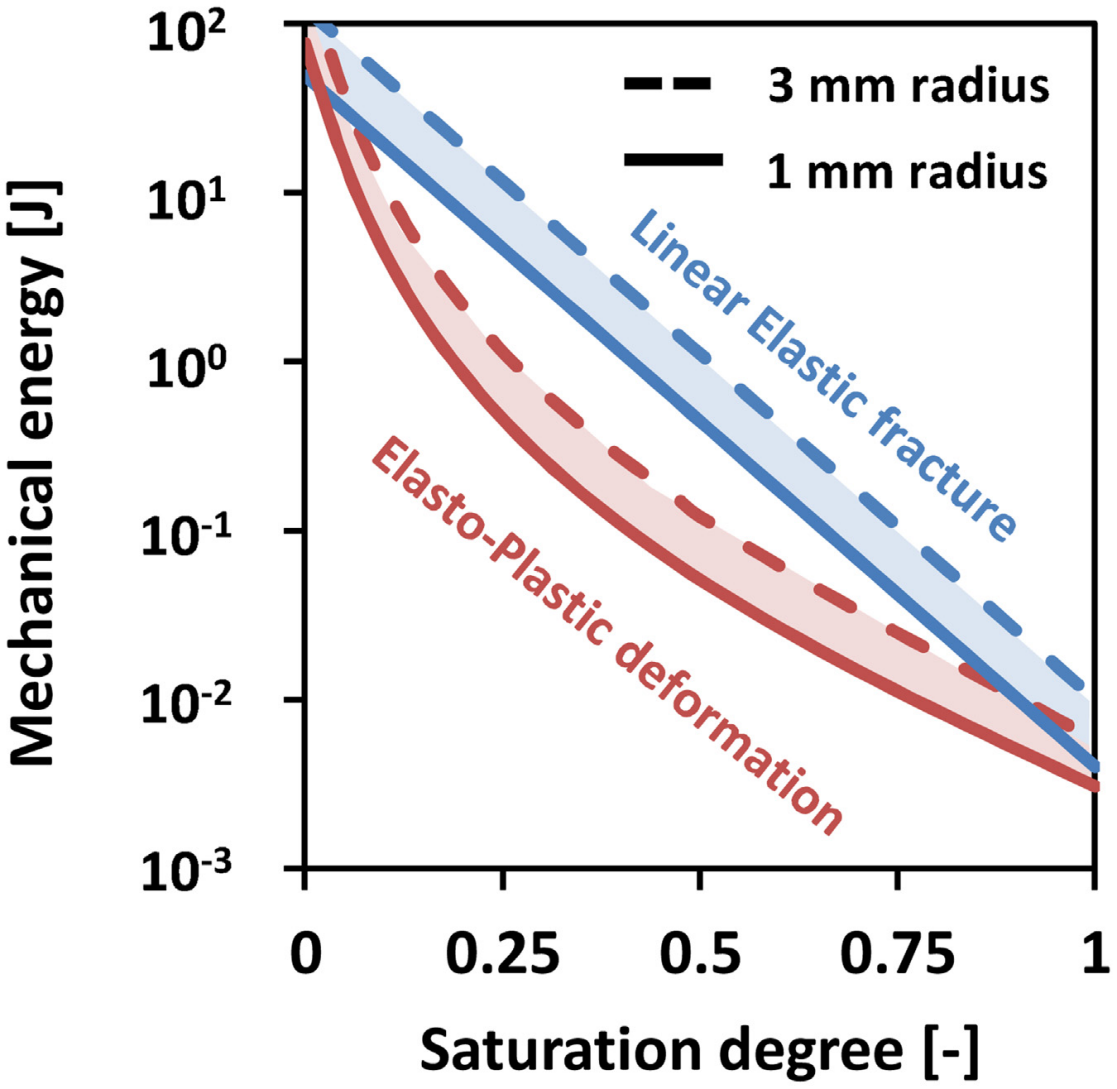
Mechanical energy to create a burrow of 1 m length and 1.2 mm radius as a function of normalized water content using a penetration model and a fracture model. Both models were conducted for a worm of r = 1.2 mm [50] for soils with clay contents ranging from 15–25% [21, 50–52].

A comparison of energy requirements for creating a 1 m long cavity with a radius of 1.2 mm by plastic penetration (our model) or fracture propagation (model by Dorgan *et al*. [50]) is presented in Fig 13. For a normalized water content of 1 [-], the crack propagation model predicts an energy expenditure of 3.2 × 10^−3^ J, while the elasto-plastic cavity expansion model presented in this study yields an energy expenditure of 3.1 × 10^−3^ J. For normalized water contents in the range of 0.2 to 1 [-], the strain energy required for penetration based on the elasto-plastic penetration-cavity expansion model is much lower than for fracturing. For normalized water contents below 0.02 [-], the strain energy required for plastic penetration exceeds that of the crack propagation.

## Discussion

The theoretical estimates and their general agreement with limited experimental data inspire confidence in using these approximations to estimate the energy requirements for creation of earthworm burrows or plant root channels. The resistance stresses for plant roots and earthworms are reduced with increasing radius (Fig 11 (a)), hence the mechanical energy per unit of displaced soil volume decreases with increasing radius. This implies that larger tunnel radii would be more energy efficient if displacing soil material where the primary goals of earthworms and plant roots. However, if movement through soil in search for resources drives bioturbation, the mechanical energy required to traverse a certain distance in the subsurface may be ecologically more relevant for plant roots and earthworms. The results in Fig 11 (b), depict an increase in penetration force with increasing cavity radii, and the mechanical energy per distance travelled in the soil increases with radius. Hence, while smaller earthworms or plant roots are less energy-efficient in terms of volumetric soil displacement, worms or roots with smaller radii are more energy-efficient for traversing the subsurface.

A decrease in strain energy for wetter soils is attributed to the reduction in soil strength and shear modulus with increasing water content. The strain energy requirements increase with increasing soil clay contents that affect soil strength and shear modulus. There is a slight increase in penetration resistance with increasing semi-apex insertion angle. The axial component of forces acting normal to the face of an earthworm or plant root increases with increased apex angles resulting in an increase in penetration resistance.

The plastic earthworm penetration model presented in this study compared well with the linear elastic fracture mechanics (LEFM) model of Dorgan *et al*. [50] at high water contents, predicting a mechanical energy expenditure of 3.1 × 10^−3^ J, whereas Dorgan *et al*. [50] estimates 3.2 × 10^−3^ J for an earthworm to propagate a crack of the same length. Despite many differences in the modeling approaches and underlying assumptions, the mechanical energy computed for crack propagation and penetration-expansion are similar for water contents near saturation [50].

For intermediate water contents, the LEFM model typically results in a larger expenditure per unit of cavity length than the plastic penetration model, while for very low saturation degrees (below 0.02 [-]), fracture propagation appears to be less energy consuming than plastic penetration (Fig 13). However, LEFM would imply that creation of permanent fractures at saturation degrees below 0.4 [-] would require cavity pressures in excess of 230 kPa, which is the physiological limit of earthworms. The results suggest that plastically deforming the soil is probably more energy efficient for wet soils, while for dry soils (saturation degrees lower than 0.02 [-]), Fig 13 suggests that crack propagation is energetically advantageous over penetration-cavity expansion. These preliminary results suggest that different modes of soil penetration may be favorable for different soil water contents (or soil mechanical properties).

Plant roots have been observed to expand radially upon encountering soils with high mechanical impedance, and in this way weakening the forefront [15], potentially inducing a brittle failure close to the root tip, allowing for axial elongation into an open crack. For the case of this soil, the water contents where linear elastic fracture mechanics seems more energetically feasible than elasto-plastic deformation is below the physiological limit where plant roots can actually uptake water (corresponding to a saturation degree of 0.2 [-]), that is the matric potential of the soil exceeds the osmotic pressures that plant roots can extract water from the root soil interface (root suction pressures reported to range from −2.5 to −3.5 MPa [54]). It has been shown that soil plastic deformation and crack propagation are not mutually exclusive phenomena (Yoshida and Hallett [55]), and we note that more systematic studies are required to delineate respective envelopes of applicability of fracture mechanics and plastic deformation models. Despite neglecting potential crack formation, the elastic-perfectly plastic model presented in this study still determines a minimum energy requirement necessary for bioturbation.

The model developed in the present study could be used to assess the amount of soil organic carbon (SOC) necessary to support earthworm activity in soil. Fig 12 depicts estimates for the minimum amount of SOC required for an individual earthworm to satisfy the energy demand of soil penetration (for clay contents of 16% and 50%). Based on these model estimates, earthworm penetration in soil with normalized water contents below 0.25 can only be economical if SOC content exceeds 10 kg m^−3^. This value seems realistic, as Crumsey *et al*. [56] also found 10 kg m^−3^ SOC in their mesocosm experiments, which were maintained at field capacity during the experiments. In order to provide conservative estimations of energetic costs of cavity creation, our analysis presented in the results section was based on normalized water content at field capacity (Θ = 0.4).

Fig 12 implies that the energetic costs for constructing a 1 m long tunnel by an earthworm with 2.5 mm radius [3, 42] at a normalized water content of 0.4 and 16% clay content are equivalent to the energy contained in roughly 2.3 × 10^−5^ kg soil organic carbon. Considering an average earthworm maintenance respiration rate at rest of 3.6 × 10^−6^ kg day^−1^ (of carbon) [57], this implies that the strain energy required to burrow 1 m distance is equivalent to 7 days of maintenance respiration at rest. Provided the upper limit of earthworm penetration rate is 0.2 m day^−1^ (Table 3) this would suggest that soil penetration could account for up to half of an earthworm’s energy budget.

Scaling up of our energy estimates to the burrowing activity of a typical earthworm community in a paddock resulted in energy expenditures equivalent to as much as 0.05 kg year^−1^ SOC per m^2^ surface area. Alban and Berry [58] witnessed invasive earthworms depleting SOC at a rate of 0.06 kg year^−1^ per m^2^, suggesting that our estimates are in close agreement with field observations. Considering that, Beer *et al*. [59] estimated the net primary productivity (NPP) in cropland to be in the order of 0.9 kg m^−2^, the estimates above suggest that earthworm populations could consume as much as 5% of the annual NPP in crop lands. Taking the lowest normalized water content for which soil penetration would still be economically feasible with an SOC concentration of 10 kg m^−3^ (Θ = 0.25 in Fig 12), a similar calculation would yield that the earthworm community energy expenditure for the same burrowing activity would be equivalent to as much as 0.15 kg year^−1^ SOC per m^2^, which would be equivalent to 15% of the annual NPP in crop lands. However, note that these estimates are based on constant soil water content, whereas natural hydration dynamics would affect the energetic costs of soil penetration as well as bioturbation activity and earthworm population sizes.

An important aspect not considered in this analysis is the role of soil ingestion by earthworms, an essential process for extracting the required soil organic carbon and contributing to many soil regulatory functions [60]. Ingested soil passing through the earthworm gut stimulates microbial activity, enhances aggregation by mucus secretion and litter collection, and gives rise to mutual interactions between microbes and earthworms. On the other hand, earthworms ingest a wide variety of organic matter including fungus and bacteria [60, 61]. Evidence suggests beneficial impact on plant root activities (despite anecdotal evidence for earthworms ingesting living roots [61]). The partitioning between ingestion and soil displacement is not well understood. Future studies will endeavour to elucidate the mechanical ramifications of ingestion, and, equally important, the role of rapid penetration by earthworms in comparison to slow growth by plant roots that would incur different energetic costs (for the same burrow geometry) due to effects of visco-elastic displacement processes [21] not addressed in this study.

Finally, the results presented in this study suggest that biotic soil processes perform considerable mechanical work on soil, implying that they are likely effective contributors to soil structural restoration after disturbance, e.g. by soil compaction. Abiotic processes such as wetting and drying induce shrinking and swelling [62], which also contributes to expanding cavities. Spherical soil aggregates undergo coalescence due to wetting at axial strain rates of 0.005 m m^−1^ s^−1^, which is equivalent to the rate of spherical radial expansion due to aggregate swelling [62]. Plant roots expand cavities at strain rates up to 0.08 m m^−1^ s^−1^ [23]. This would imply that macropore creation by biotic processes occurs at rates nearly 20 times faster than that by abiotic processes, thus earthworms and plant roots have the capacity to accelerate soil structural restoration by at least an order of magnitude compared to abiotic processes only. More experimental and theoretical work is needed to understand the interplay between abiotic and biotic processes during post-compaction soil structural restoration. In particular, investigation of the initiation of the process, i.e. the creation of the first channels or cracks, and their benefit for subsequent colonization by roots and earthworms is important, in order to enable quantitative prediction of the speed of recovery of compacted soils.
