# Correction: Soil Penetration by Earthworms and Plant Roots—Mechanical Energetics of Bioturbation of Compacted Soils

**DOI:** 10.1371/journal.pone.0136225

**Published:** 2015-09-01

**Authors:** The PLOS ONE Staff

Fig 13 is incorrect. The authors have provided a corrected version here.

**Fig 13:**
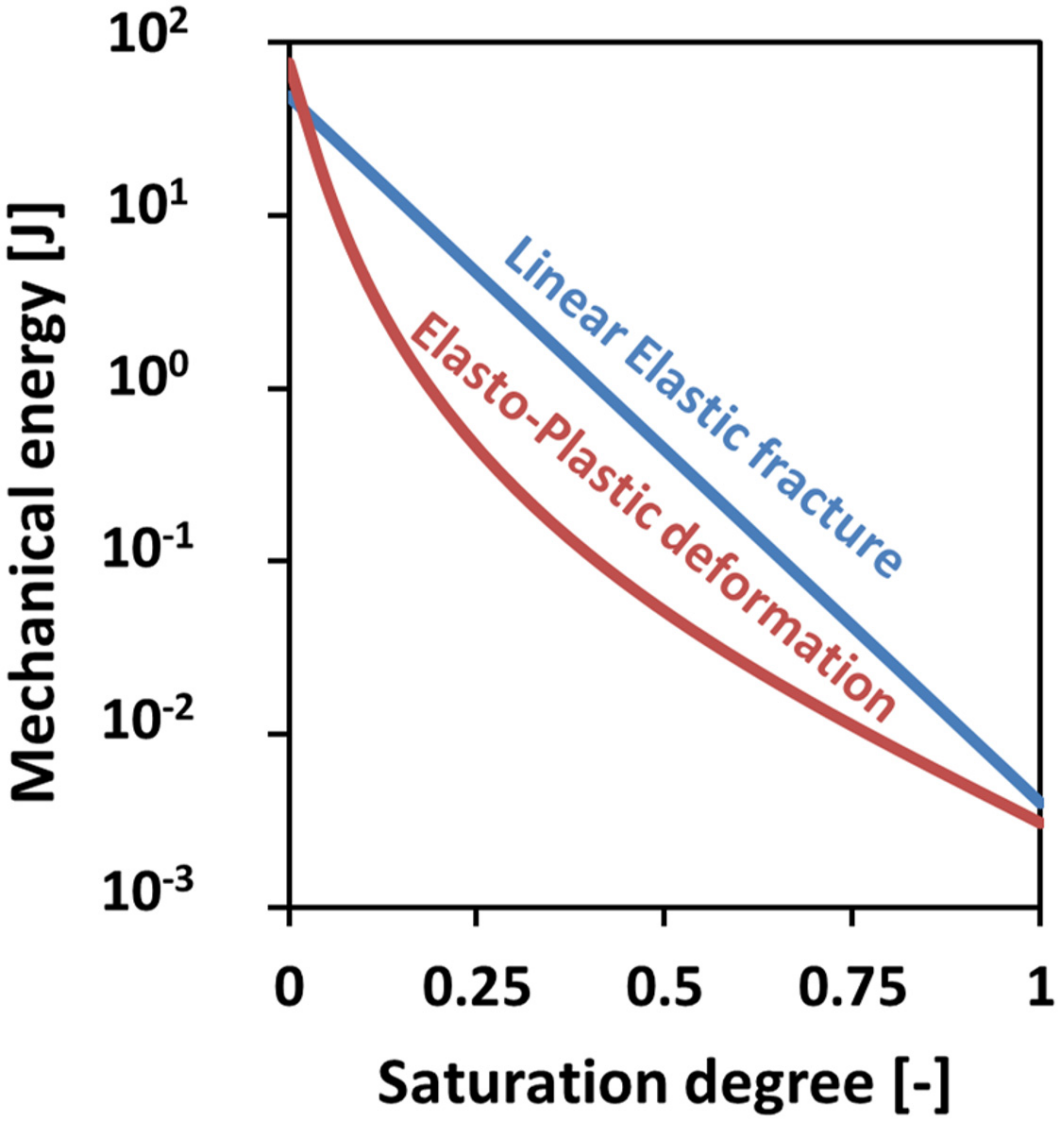
Mechanical energy to create a burrow of 1 m length and 1.2 mm radius as a function of normalized water content using a penetration model and a fracture model. Both models were conducted for a worm of r = 1.2 mm [50] for soils with clay contents ranging from 15–25% [21, 50–52].
